# Expanding the prevention armamentarium portfolio: A framework for promoting HIV-Conversant Communities within a complex, adaptive epidemiological landscape

**DOI:** 10.1080/17290376.2015.1034292

**Published:** 2015-04-17

**Authors:** Christopher J. Burman, Marota Aphane, Oliver Mtapuri, Peter Delobelle

**Affiliations:** ^a^ PhD, is a senior lecturer with the Rural Development and Innovation Hub, University of Limpopo, Turfloop Campus, Polokwane, South Africa; ^b^ Master of Development, is a research assistant with the Rural Development and Innovation Hub, University of Limpopo, Turfloop Campus, Polokwane, South Africa; ^c^ PhD, is a senior lecturer with the Turfloop Graduate School of Leadership, University of Limpopo, Polokwane, South Africa; ^d^ MD PhD, is a senior lecturer with the School of Public Health, University of the Western Cape, Cape Town, South Africa

**Keywords:** chronic schemas, complex adaptive epidemiological landscape, disruptive innovation, pattern management, safe-fail probes, schémas chroniques, contexte épidémiologique complexe adaptif, innovation disruptive, gestion des schémas, expérimentation à sécurité intégrée

## Abstract

The article describes a design journey that culminated in an HIV-Conversant Community Framework that is now being piloted in the Limpopo Province of South Africa. The objective of the initiative is to reduce the aggregate community viral load by building capacity at multiple scales that strengthens peoples' HIV-related navigational skill sets—while simultaneously opening a ‘chronic situation’ schema. The framework design is based upon a transdisciplinary methodological combination that synthesises ideas and constructs from complexity science and the management sciences as a vehicle through which to re-conceptualise HIV prevention. This resulted in a prototype that included the following constructs: managing HIV-prevention in a complex, adaptive epidemiological landscape; problematising and increasing the scope of the HIV knowledge armamentarium through education that focuses on the viral load and Langerhans cells; disruptive innovation and safe-fail probes followed by the facilitation of path creations and pattern management implementation techniques. These constructs are underpinned by a ‘middle-ground’ prevention approach which is designed to bridge the prevention ‘fault line’, enabling a multi-ontology conceptualisation of the challenge to be developed. The article concludes that stepping outside of the ‘ordered’ epistemological parameters of the existing prevention ‘messaging’ mind-set towards a more systemic approach that emphasises agency, structure and social practices as a contribution to ‘ending AIDS by 2030’ is worthy of further attention if communities are to engage more adaptively with the dynamic HIV landscape in South Africa.

## Résumé

L'article porte sur un modèle de communauté touchée par le VIH dans la province de Limpopo en Afrique du Sud. L'initiative conduite dans cette communauté a pour objectif de réduire la charge virale collective en renforçant, à de multiples échelles, les facultés de navigation des individus en relation au VIH, tout en ouvrant un schéma de « situation chronique ». Le modèle est basé sur une approche transdisciplinaire méthodique qui synthétise des idées et concepts des sciences de la complexité et des sciences de gestion, de façon à ré-conceptualiser la prévention du VIH. Cela permettait de développer un modèle incluant les concepts suivants : la gestion de la prévention du VIH dans un contexte épidémiologique complexe adaptif; approche méthodique et large de l'arsenal de connaissance du VIH par le biais de l'éducation ciblée sur la charge virale et les cellules de Langerhans ; innovation disruptive et de l'expérimentation à sécurité intégrée conduisant à une dynamique de création, et de la mise en œuvre des méthodes de gestion des schémas. Ces notions sont sous-tendues par une approche préventive ‘intermédiaire' conçue pour couvrir la prévention par ‘faille’, permettant de développer une conceptualisation multi-ontologique du défi intéressé. En conclusion, l'article recommande de dépasser les paramètres épistémologiques conventionnels existants en matière de prévention, au profit d'une approche plus systémique, soulignant l'action, la structure et les pratiques sociales, afin de contribuer à l’éradication du SIDA d'ici 2030, pour autant que les communautés soient prêtes à adopter un comportement mieux adapté au contexte dynamique du VIH en Afrique du Sud.

## 1. Introduction

This article describes a conceptual, prototype framework that has been developed in the Limpopo Province of South Africa which was named ‘promoting HIV-Conversant Communities’. The design was catalysed through a community–university partnership between the Waterberg Welfare Society (WWS) and the Rural Development and Innovation Hub, University of Limpopo. The framework was a response to concerns by community health-care workers that some of the challenges they experience are recurring issues that seemed impossible to overcome—including disclosure, adherence to medication and loss to follow up.

In response to this, the university developed a prototype framework influenced by complexity science and the management sciences. The overall objective of the initiative is to reduce the aggregate viral load in the community and the partnership agreed that the purpose of the collaboration would be to transform the prototype into a pragmatic intervention that can be replicated at scale. Since the inception of the initiative in 2013, two other study sites have become operational in the Mopane District of Limpopo—Pfukani home-based carers near Letsitele and a commercial avocado farm called Westfalia close to Tzaneen.

The framework design was originally influenced by the South African National Strategic Plan on HIV, TB and STIs, 2012–2016 (NSP) which called for the ‘development of innovative new approaches for the prevention, diagnosis, treatment and care, and mitigation of the impact of HIV, STIs and TB, either singly or in combination’ that are inclusive of ‘different combinations of interventions … designed for the different key populations’ (SANAC 2012:70, 14). It also has resonance with a recent initiative by UNAIDS to ‘end AIDS by 2030’ which reinforces the need for innovation: ‘a business-as-usual approach or simply sustaining the AIDS response at its current pace cannot end the epidemic’ UNAIDS (2014:296). This global re-orientation is being promoted because there have been more biomedical ‘achievements in the past five years than in the preceding 23 years’ which means that ‘there is hope that ending AIDS is possible’ (UNAIDS 2014:296). UNAIDS argue that ‘ending AIDS’ requires a shift from the existing ‘response mode’ to a ‘mission mode’ that requires eight action points. The prototype responds to five of these action points, including ‘(1) Protect[ing] human rights, embrac[ing] the human family and leav[ing] no one behind; (2) Investing in communities; (4) Focus[ing] on local epidemics and populations’; ‘(6) Expanding choices for HIV prevention and treatment’ and ‘(7) Integrat[ing] HIV programmes with other health and development programmes’ (UNAIDS 2014:299–302).

The prototype framework, comprising three principle components—education, experiential application of the learning and implementation—was designed with the ambition of opening innovative ‘middle-ground’ intervention spaces that better equip rural communities to construct novel and contextually relevant skill sets to assist them to navigate the complex, adaptive epidemiological HIV landscape. Navigation of this landscape is both a prevention and treatment challenge and—increasingly—a chronic disease management challenge (Chu & Selwyn 2011).

The practical focus of the framework is to enable people to begin to modify their mental models, or schemas (Schon 1984), so that their old mental models about the epidemic are destabilised as a precursor to enabling them to improve their navigational abilities in ways that reduce exposure to risks that can facilitate the movement of the virus from one body to another—thereby contributing to community viral load reduction.

A distinction was made between a primary and secondary ambition within the framework. The primary ambition was to initiate a shift from an exclusive focus on prevention towards the notion that HIV infection is a phenomenon that can be *acquired*, *facilitated* or *transmitted*. The reason for emphasising the interrelated elements of transmission is that a 30-year-old epidemic, that has affected practically every community in South Africa, can no longer be confronted exclusively through a ‘prevention’ lens—as in the 1980s/1990s—if framed from the perspective of reducing the aggregate community viral load (Ellman 2015).

The secondary ambition was to emphasise that the epidemic is a ‘long-wave event’ (Fourie & Follér 2012:255) which can now be characterised as much by a ‘chronic’ schema as a ‘prevention’ schema. This ambition was influenced by the recognition that in South Africa there is a ‘generalised HIV epidemic’ (NSP 2011:13) representing a chronic situation that requires dynamic management—to ‘take HIV from an epidemic to a manageable disease’ (Dybul 2014).

It is hoped that the primary ambition will result in the development of management practices that enable locally relevant, practical skill sets that can be applied within the pilot sites—and potentially beyond—and that the secondary ambition will develop ‘anticipatory thinking’ that begins the process of preparing people for the processes of managing dynamic, chronic situations (Edgman-Levitan, Brady & Howitt 2013).

**Fig. 1.**
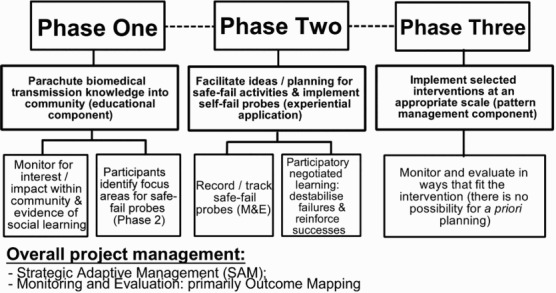
Framework design overview.

*Source:* Authors’ contribution.

Strategic Adaptive Management (SAM) was applied as the overarching management tool (Pade-Khene, Luton, Jordaan, Hildbrand, Gerwel Proches, Sitshaluza, *et al.* 2013) and Outcome Mapping as a practical guide for monitoring and evaluation (Van Ongevalle & Peels 2014)—alongside more traditional forms of academic reporting. The overall framework design is presented in Fig. 1.

While we recognise that many of these approaches represent unusual angles from which to tackle an HIV intervention, the ambition is to develop a paradigm shifting experience that expands the existing prevention armamentarium and contributes to the ambition of ‘ending AIDS by 2030’.

In the sections that follow we describe the design journey before bringing the component parts together into an action-oriented methodological statement. We do so by introducing the frame that influenced the overall design—HIV as a complex adaptive epidemiological landscape and a ‘middle-ground’ intervention; four constructs that we have transposed into the discourse of HIV and finally the overall ambition of the framework that emerged through the iterative design process.

## 2. Re-framing the HIV challenge into a complex adaptive epidemiological landscape

### 2.1. Adaptive

The expression ‘adaptive’ is often understood to mean that something, such as a particular species, adapts to certain environmental conditions. The way in which we apply ‘adaptive’ takes the position that through the process of its existence the species' interactions discretely affect the environment—as well as adapt to it. This represents an interdependent relational process of change—not a one-way conceptualisation of evolution (Levins & Lewontin 1985). In this formulation, adaptive characteristics are co-evolutionary in the sense that a particular development, or change, is facilitated by relationships with other dynamics within the system—which, in turn, influence the characteristics of the agents within the system—as well as having potentials to influence system-level properties (Anderson, Crabtree, Steele & McDaniel 2005).

This conceptualisation is associated with complexity science and has gradually been drawn into multiple disciplines—based on the fundamental premise that agents act and influence their environment in the process of adaptation—and this somewhat unpredictable, nonlinear combination influences the future trajectory of history (Ramalingham 2013).

### 2.2. Complex

HIV prevention is often framed as a ‘problem’ that can be ‘solved’ based on the presumption that traditional Newtonian ‘rationality’, within which ‘cause and effect’ is omnipresent, will necessarily result in a solution being definable and within reach. In the sphere of HIV, there are critical concerns about this assumption (Stillwaggon 2012). In contradistinction to assuming traditional science always has the capacity to identify the ‘problem’—and hence locate an ‘answer’—we take the lead from Piot, Bartos, Larson, Zewdie and Mane (2008) who emphasised that the HIV landscape is ‘complex’, and, hence, suggest that it is legitimate to frame HIV *transmission, facilitation* and *acquisition* as ‘wicked problems’ (Rittel & Webber 1973).

It is claimed that ‘wicked’, or complex, intractable problems—such as those experienced by the community health-care workers at WWS—demonstrate qualitatively unique characteristics which ‘refer to the nature of the problem not the degree of difficulty’ (Stirzaker, Biggs, Roux & Cilliers 2010:600). Specifically, these types of challenges contain some nonlinear characteristics and the ‘non-linear interactions and feedbacks between components give the system a degree of unpredictability. Cause and effect can usually be resolved when looking backward, but one cannot be sure which of several logical outcomes will eventuate when looking forward’ (Stirzaker *et al.* 2010:600). These types of challenges have repeatedly presented dilemmas which have confounded traditional Newtonian approaches to problem-solving (Ramalingham 2013; Weaver 1948).

Modelling epidemics is a good example of the challenge of predicting the future in such scenarios:

Modelling studies are a mixed blessing. Models can generate useful data if they are based on realistic assumptions and up to date biomedical data. However, this often proves difficult, especially for long-term analyses, because even small inaccuracies or *other unforeseen changes* may lead to very different results. (emphasis added, Whiteside & Strauss 2014:107)

Not only does a complexity lens caution that in complex situations it is unrealistic to expect that concrete ‘solutions’ to ‘problems’ can be identified in advance; it also broadens the analytical scope by placing great emphasis on ‘understanding the *patterns* of relationships among its agents’ (emphasis added, Anderson *et al.* 2005:672). Of particular relevance is the way in which the influence of ‘agents interacting in a non-linear fashion may [unexpectedly] self-organize and cause [new] system properties to emerge’ making prediction, based on Newtonian presumptions, elusive (McDaniel & Driebe 2001:19; also see Ramalingam, Jones, Reba & Young 2008).

In an unpredictable context—or a ‘causal thicket’ (Wimsatt 1994)—it is ‘futile’ to adhere to the traditional rules of the Newtonian game (Wilson, Holt & Greenhalgh 2001). In a complex situation it is necessary, and legitimate, to step outside of the Newtonian box as a mechanism for addressing this type of challenge and work with the imperfect knowledge at hand.

This complexity is recognisable in South Africa where 6.4 million people are known to be living with HIV (Shisana, Rehle, Simbayi, Zuma, Jooste, Zungu, *et al.* 2014) which means that it is now a ‘long wave event’ that requires the management of over six million—increasingly lengthy—lives to be negotiated as treatment opportunities expand (Bor, Herbst, Newell & Barnighausen 2013). In order to adapt to this emergent, qualitatively unique, context, the effective management of HIV requires some bold—yet justifiable—exploratory steps outside of the Newtonian box. Based upon the premise that it is sometimes legitimate to step outside of the Newtonian box, we now consider the ‘middle-ground’ theorem that influenced the prototype design.

### 2.3. Why ‘middle ground’?

During the prototype design the university component of the partnership focused upon adopting what has been labelled the ‘middle ground’ between agency and structure. The expression ‘middle ground’ is a deliberate metaphor used to reiterate that both individual agency *and* social structure influence the introduction of new social practices (Kippax, Stephenson, Parker & Aggleton 2013). Kippax *et al.* (2013) argue that the ‘vulnerability’ approach to HIV prevention over-emphasises the role of individual agency in change processes and that the ‘resilience’ approach over-emphasises structure—hence their call for a ‘middle ground’ that focuses on the indeterminate combination of both. Specifically they argue that ‘people act (agency) to transform the social (structure) via practices that they develop to respond to … HIV risk’ (Kippax *et al.* 2013:1370). We concur that both agency and structure influence social change and propose that complexity science provides a vehicle through which to incorporate the ‘middle ground’ into an intervention design.

#### 2.3.1. Complexity, the middle ground and the intervention design

There is a growing body of literature with regard to health and complexity—for example, see Jayasinghe (2011) and Paina and Peters (2012)—but there is little in the way of transferring theory into practice, especially in the domain of HIV in rural South Africa (Gilson, Elloker, Olckers & Lehmann 2014). The ‘middle-ground’ metaphor was used to guide the design process as the first step towards closing the gap between theory and practice—that is inclusive of both agency and structure—and developed into two key ambitions.

1. The first ambition was to emphasise—from a grassroots perspective—the importance of the ‘middle ground’ in an intervention designed to reduce the aggregate viral load. The ambition aimed to emphasise that (a) individuals can reduce the risk of *acquiring, facilitating or transmitting* the virus from one body to another—thus influence the aggregate viral load and (b) while the environment (structure) influences the aggregate viral load, people within communities have the potentials to influence the lived environment—for example, by encouraging the use of soap and personal hygiene within households, thus influencing co-infections and the aggregate viral load.
  The purpose of this focus was to facilitate awareness that the opportunities afforded by recent biomedical breakthroughs represent opportunities that rural communities can exploit—at both an individual (agency) and a structural level. This ambition developed because—despite the qualitatively radical epidemiological shift into a new era of biomedical opportunities—the tempo at which ‘achievements’ are being to responded has been diverse, fragmented, and uneven, often contingent on political, economic and legal influences, as well as individual agency (UNAIDS 2014). However fragmented the implementation of these opportunities has been; they still remain opportunities that are relevant to reducing the aggregate viral load—thus relevant to the prototype design.
2. The second ambition included surfacing and incorporating localised sentiments—or ‘sensori-memorabilia’ (Burman, Mamabolo, Aphane, Lebese & Delobelle 2013:22)—that have emerged from affected communities' interactions with the epidemic. The reason for surfacing these historically constructed, heterogeneous, and often embodied sentiments is that they are sometimes shrouded in secrecy (Stadler 2011); yet they represent essential—even if discrete—structural phenomena that contribute to ‘knowing’, and working with, ‘our epidemic’ (Wilson & Halperin 2008). Following Dickinson (2013), the ambition of incorporating these sentiments into the framework was because their influence is as relevant as that of formal knowledge and material realities as critical mediators of the future trajectory of the epidemic. Further to this, while community ‘sensori-memorabilia’ is a structural influence of the trajectory of the epidemic, it is also something that communities can change.

The ‘middle-ground’ metaphor was included into the prototype as a reminder that while many seemingly abstract factors—many of which demonstrate complex characteristics—it is possible for communities to influence both the structural determinants, while simultaneously adopting risk-reduction social practices at a more proximal level. It is from this platform that we turn to the conceptual details of the framework.

## 3. The four constructs

### 3.1. Construct one: managing HIV-prevention in a complex, adaptive epidemiological landscape

We introduce this notion based upon a concern that there is an ‘epistemic fault line’ in the realm of HIV—not only at the meta-theoretical level (Adam 2011)—but also at the level of implementation.

We have suggested that managing—or responding to—HIV is a complex challenge because some of the relationships are nonlinear, making elements of the HIV landscape unpredictable (Burman, Aphane & Delobelle forthcoming). This means simultaneously negotiating both the ordered Newtonian world of ‘cause and effect’ and the unordered world of complexity. In order to guide the framework design and the subsequent interventions, a multi-ontology management heuristic called the Cynefin framework was applied (Snowden 2005; Van Beurden, Kia, Zask, Dietrich & Rose 2013). The Cynefin framework is a heuristic designed to ‘help leaders determine the prevailing operative context so that they can make appropriate [management] choices’ (Snowden & Boone 2007:4).

**Fig. 2.**
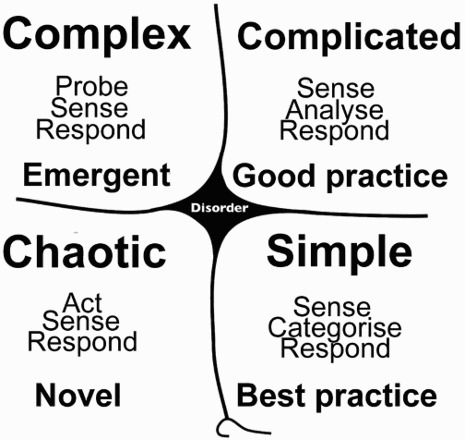
The Cynefin framework.

Source: Adapted from http://en.wikipedia.org/wiki/Cynefin.

The framework is made up of four quadrants and split between ordered and unordered domains. The ordered domains are the territory of Newtonian science and the unordered domains represent spaces where ‘there is no immediately apparent relationship between cause and effect’ (Snowden & Boone 2007:71). The fifth domain is called ‘disorder’. Disorder ‘applies when it is unclear which of the other four contexts is predominant’ (Snowden & Boone 2007:69) (Fig. 2).

The Cynefin framework was originally designed to ‘help executives sense which context they are in so that they can not only make better decisions but also avoid the problems that arise when their preferred management style causes them to make mistakes’ (Snowden & Boone 2007:69).

From the perspective of HIV, the context of the biomedical world is fundamentally—and legitimately—rooted in the Newtonian legacy represented by the ‘ordered’ domain. The naturalistic, real-world, contexts of interventions that exploit opportunities enabled by biomedical breakthroughs—such as adherence to medication or consistent condom use—reside within the ‘unordered’ domains.

The purpose of applying this heuristic was to guide the implementation of the HIV-Conversant Community Framework so that the responses applied during the interventions correspond with the appropriate decision-making domain. The reason for emphasising the appropriate management response to different challenges is because some implementation strategies are inadvertently conceptualised as activities situated within ordered contexts, when they demonstrate ‘unordered’ properties (Florczak, Poradzisz & Hampson 2012). We concur with Adam (2011:2) that ‘HIV prevention need not be an either/or choice between competing or antagonistic knowledge systems’ and the Cynefin framework is included as an implementation heuristic to close the ‘epistemic fault line’ because appropriate management responses to the challenges identified in the pilots will likely be a critical mediator of outcomes of the initiative.

### 3.2. Construct two: problematising and increasing the scope and potentials of the HIV knowledge armamentarium

The overriding concern while considering the role of education within the framework was that the information should be comprehensive and up to date so that the second, experiential application, phase would enable opportunities for ‘everyone [to] understand, through the *personalisation* of information, the risk of acquiring HIV’ (emphasis added, Shisana *et al.* 2014:xxxix). The educational platform was thus required to achieve three principle objectives:

1. To increase the scope of knowledge about HIV *acquisition*, *facilitation* and *transmission*;
2. To begin a process of critically interrogating existing practices and imagining future practices to reduce HIV *acquisition, facilitation or transmission*; and
3. To begin a process of sensitising people to HIV as a chronic management issue.

In order to begin to emphasise the importance of ‘personalising’ information about the acquisition, facilitation and transmission of HIV, environmental factors that enable co-infections to thrive was used as an educational heuristic. The justification for using this heuristic is that high population-level viral loads in Southern Africa may be influenced by the ‘the high burden of co-infections’ (Abu-Raddad, Barnabas, Janes, Weiss, Kublin, Longini, *et al.* 2013:981) making environmental co-infections a relevant educational device that could promote the notion of ‘personalising risk’. It was planned that heuristic be applied in the following way:

1. In much the same way that other chronic conditions can be co-managed by patients, HIV-infected individuals, and their affected networks, can influence some factors that influence the trajectory of the chronic condition—if they have appropriate and relevant knowledge about the factors that influence disease progression;
2. From a community perspective, existing social practices contribute to the existing community aggregate viral load and there is the possibility that new knowledge about the influence of these practices will catalyse community mobilisation to alter these practices—hence contribute to reducing the aggregate viral load. For example, a child who picks up a stomach infection because there is no soap at school can introduce that infection into a community beyond the school gates. For HIV+ people in that community the stomach infection may develop into a co-infection that facilitates an increase in the aggregate viral load (Greenland, Cairncross, Cumming & Curtis 2013; Modjarrad & Vermund 2010). Awareness of the *connections* between soap in schools, co-infections and the aggregate viral load could become a catalyst to alter the frame through which people perceive wellness in the era of HIV. In turn this has the potential to contribute to altering existing social practices and thus becomes a useful educational heuristic to critically examine the collective influence of communities in shaping the current and future trajectory of the epidemic;
3. In order to open the chronic schema space, the issues cited above are applied as discussion points so that ‘anticipatory chronic literacy’ is conceptually linked to both structural and proximal forms of risk.

The intention of this construct is to provide opportunities for the participants to begin to critically interrogate how to apply the new biomedical information in multiple ways. Throughout the process of transforming the prototype into an implementable strategy other educational heuristics will be identified and documented.

The focus of this aspect of the design was to emphasise that there are community responses that are imaginable—and thus potentially implementable—that can contribute towards the ambition of ‘ending AIDS’.

#### 3.2.1. The educational package: A-3B-4C-T

The educational package—A-3B-4C-T—that was agreed upon was accredited with the South African Health Care Professions Council in 2014 and focuses on risk-reduction and prevention. It highlights the way in which the HIV virus is transmitted using two educational heuristics—Langerhans cells and the viral load—to weave the curriculum together. The curriculum includes 9 primary strategies that can reduce HIV transmission as stand-alone interventions and 25 associated risk-reduction mechanisms.

The nine strategies include: A = antiretrovirals (treatment as prevention); B1 = barriers (physical barriers such as condoms, and behavioural barriers such as abstinence and partner reduction); B2 = babies (the Department of Health Prevention of Mother to Child Transmission protocols); B3 = blood and breached skin and membrane precautions and protocols; C1 = co-infections (infections that accelerate HIV disease progression); C2 = circumcision (specifically, voluntary male medical circumcision), C3 = community viral load management (primary, occupational and environmental health issues impacting upon immune function and viral load), C4 = couples (couples' HIV counselling and testing (HCT)), and T = testing (HCT itself, as well as issues of transmission during the window period)—(adapted from Leadership & Training Resource Centre 2014).

The educational package takes this high level of detail to a manageable level of cognitive abstraction—through the Langerhans cells and viral load heuristics—providing opportunities for the learners to construct new mental models about the virus–human–environment relationships which can be applied to multiple HIV-related challenges.

### 3.3. Construct three: disruptive innovation and safe-fail probes

#### 3.3.1. Disruptive innovation

We transpose the concept of ‘disruptive innovation’ from the private sector (Christensen 1997); but do so in a metaphorical, rather than, a literal sense—while acknowledging that the concept has only occasionally been applied to health-related concerns (Bevan & Fairman 2014; Jameson 2014).

Christensen (1997), kick-started a debate about innovation types in a rapidly changing world. The essence of this discussion is as companies compete for market share they are often faced with unexpected external forces, or shocks—typically other competitors' products, new laws, or a shift in demand—which have the potential to reduce the bottom line. The ‘dilemma’ that the company is faced with is whether to continue to attempt incremental improvements in their existing products or to opt for a qualitatively different ‘game-changing’ option (Jameson 2014:2822). A recent example of the ‘innovator's dilemma’ in the sphere of HIV is the initiative to make condoms more attractive to students in South Africa (SAPA 2014) as a response to findings that between 2008 and 2012 condom use ‘significantly decreased’ (Shisana *et al.* 2014:xxxiv). This decision seems to have been based upon the linear assumption that a more attractive condom will necessarily result in increased condom usage. In this instance the ‘dilemma’ is: Do we develop and promote an improved high-end condom product, or do we look for a ‘game-changing’ frame that enables ‘the unwashed masses’ (Christensen, Bohmer & Kenagy 2000:3) to take more ownership of the ‘condom fatigue’ cited in the newspaper article?

From the perspective of the management heuristic—the Cynefin framework—this signals a slightly different question: Do we make a decision based on the assumption that the uptake and appropriate use of a condom is a linear, ordered challenge and pursue more sophisticated high-end technologies; or do we temporarily consider shifting the problematic of condom use into the unordered domain of nonlinear uncertainty and respond accordingly?

From the perspective of designing a framework that makes prevention more accessible within grassroots communities—disruptive innovation provides a useful connection with the necessity to begin to alter frames that may be unintentionally restrictive—because they are fundamentally reliant on ‘ordered’ sexual behaviour change and biomedical breakthroughs—to options that increase the diversity of prevention avenues by exploiting opportunities residing within the unordered domain of complex uncertainty.

However, our concern while developing the prototype framework was that while disruptive innovation provides expansive opportunities for change, it did not fully equip the framework with implementation opportunities for grassroots innovation after the disruption process. It is for this reason that impact-oriented, experiential safe-fail probes were decided upon.

#### 3.3.2. Safe fail probes—from ideas to new social practices

One cornerstone of the framework design was to increase the diversity of actionable options that have the potential to coalesce into emergent strategies that increase the navigational skill sets of grassroots communities in complex situations. Developing actionable options in a complex situation involves ‘negotiated learning’ (Jones 2011:37–47) requiring participatory ‘deliberative processes’ with agreed objectives that emphasises the integration of ‘technical analysis and stakeholder and lay public deliberation, contrasting with the traditional ‘top-down’ or ‘bureaucratic rationalistic’ orientation’ (Lomas 2005:17). In order to contain these concepts within an implementable strategy a safe-fail design was applied.

Safe-fail experimentation generates multiple perspectives of the challenge which has the potential to produce diverse ideas that could contribute to expanding innovative opportunities for the future. Safe-fail probes provide spaces for localised experimentation which is underpinned by a coherent management strategy that values negotiated learning through failure and success. Typically 40–50% of the experiments fail but the collective learning from the process enables strategic navigational opportunities, based on the development of home-grown skill sets—grounded in localised material and embodied realities—to emerge (Snowden 2010).

The management of this process requires leadership that does not attempt to ‘impose a course of action’, but rather ‘patiently allow the path forward to reveal itself’ because in these circumstances people are unable to predict ‘a priori what [will] work. Instead, they [have] to let a solution emerge from the materials at hand’ (Snowden & Boone 2007:72). The process ‘exhibits safe failure, whereby shocks and challenges can be navigated and absorbed’ (Ramalingham 2013:211) without creating a threat to the host organisation, or community. From this process of ‘managed emergence’ consensual, horizontal decisions are taken about the strategies that have the potential to work and they become the basis for future actions, or interventions, at appropriate scales (Dickens 2012).

The potential of the safe-fail approach is that it incorporates both the technical, biomedical realm of order and the embodied, localised ‘sensori-memorabilia’ (Burman *et al*. 2013:22) of the unordered domain into the implementation design because it is undertaken by people who are familiar with the particular—and historical—idiosyncrasies of the epidemiological landscape in different localities. This universal design enables diverse, localised amelioration responses to emerge and to be purposefully incorporated into future actions. In order to facilitate this emergence, path creation and pattern management were decided upon.

### 3.4. Construct four: facilitating path creations and pattern management

#### 3.4.1. Facilitating path creations

The concept of a ‘path creation’ is a critical response to the Path Dependency literature. Path Dependency theorists argue that in some complex situations a phenomenon maintains a presence for far longer than ‘ordered’ logic would expect. The most cited example is the presence of the QWERTY keyboard—which is an inferior technology, but maintains a global presence (David 1985). Dependency theorists argue that this happens because of nonlinear feedback loops ‘increasingly restrain present and future choices’ (Koch, Eisend & Petermann 2014:67) so that the inferior technology becomes ‘locked-in’ (Roedenbeck 2011:26).

In response to this, Path Creation theorists argue that the Path Dependency theorem overstates the role of ‘structure’ over agency. Specifically, they are critical of three issues. The first is the emphasis on ‘history’. Path dependency theorisers claim that history is given and immutable. However, a counter argument is presented which emphasises that it is possible to re-frame history enabling people to ‘mobilise the past in support’ of their ambitions (Garud, Kumaraswamy & Karnøe 2010:770). The second issue is ‘contingency’—or control that actors have over structural influences. Path Creationists argue that it is possible for people to mobilise and counter some structural forces. The third issue is the ability of people to tackle feedback in the system that perpetuates the presence of the locked-in phenomenon. We concur that these feedback loops can be manipulated and explore this in more detail in the section relating to pattern management. This critique enables the Path Creationists to argue that there is always potential to influence movement within—or of—the pathway in question.

The ‘path creation’ theorem parallels the ‘middle-ground’ theorem articulated by Kippax *et al.* (2013) by emphasising that both agency and structure influence social change and these themes and the implications for the HIV-Conversant Community Framework are represented in Table 1.

**Table 1.** Path dependence vs. path creation and implications for the HIV-Conversant Community Framework.

Dimensions	Path dependent	Path creation	Potentials for the pilots
‘Initial conditions’	Given	Constructed	Can reflect selective ‘sensori-memorabilia’ of actors
‘Contingencies’	Exogenous and manifest as unpredictable, non-purposive, and somewhat random events	Emergent and serving as embedded contexts for ongoing action	Ameliorative strategies can be proactively pursued and risky activities dampened
‘Self-reinforcing mechanisms’	Given	Also strategically manipulated by actors	By including the broader community; norms and values can be promoted to reinforce individual ‘efficacy’ into more ‘distributed’ forms of collective efficacy
‘Lock-in’	Stickiness to a path or outcome absent exogenous shocks to the system	Provisional stabilisation within a broader structuration process	The emergent context both enables mitigation innovations and becomes an improved environment to live in

Source: Adapted from Garud *et al.* (2010:769).

The potential of path creations is abstract, but from the perspective of visualising the overall framework ambition it provides a vehicle through which to view both the systemic forces and the agentic activities that shape the characteristics and properties of the epidemiological landscape. It also opens an innovative analytical tool—if applied longitudinally—to track the shifts that emerge during the pilots. This ambitious monitoring and evaluation tool will be critically interrogated during the pilots.

#### 3.4.2. Pattern management

Pattern management emphasises that in the early phases of implementation of a response to a complex challenge it is impossible to precisely ‘forecast or predict [the outcome] … because the external conditions and systems constantly change’ (Snowden & Boone 2007:70). However, what does become visible through the implementation process are ‘instructive patterns’ (Snowden & Boone 2007:72) which are manageable if an overarching goal has been agreed in advance. In order to manage the emergent ‘instructive patterns', a strategy of reinforcing the preferred patterns and obstructing or dampening the unwanted ones is applied—based on regular monitoring and evaluation of the activities.

**Fig. 3.**
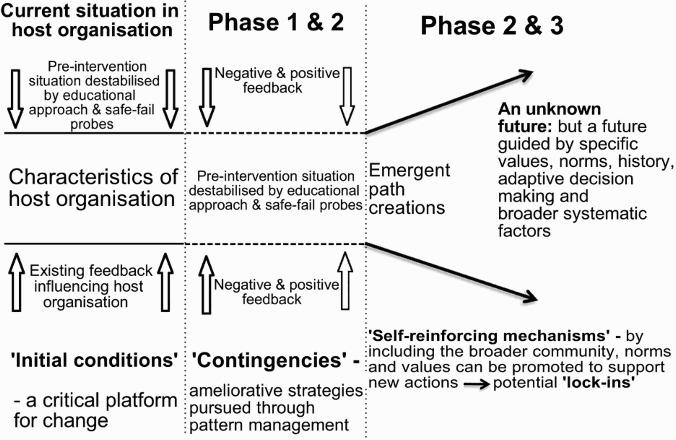
Combining path creation and pattern management within the HIV-Conversant Community Framework.

Source: Adapted from Garud *et al.* (2010:769).

By managing the overall project using Strategic Adaptive Management (SAM) and Outcome Mapping, it was hoped that locally relevant strategies could be developed and reinforced in ways that make sense in different contexts. By combining path creation with pattern management the following overview of the vision was agreed upon (Fig. 3).

It was through the process of designing this prototype framework—and many community–university discussions—that the theoretical and practical ambition of the pilots was agreed.

## 4. The Ambition of the HIV-Conversant Community pilots

### 4.1. A theoretical statement

Kippax *et al*. (2013:1373) argue that change processes are normalised through community ‘dialogue [through which] social practices are modified and other practices, such as safe sexual practices … are produced [and] norms that enable and sustain safe sex … are built’—also see Campbell, Nhamo, Scott, Madanhire, Nyamukapa, Skovdal, *et al.* (2013) and Laher, Cescon, Lazarus, Kaida, Makongoza, Hogg, *et al.* (2012) for similar arguments in southern African contexts. This argument parallels recent thinking in rural innovation studies, influenced by complexity science that promotes an evolutionary, process-oriented perspective of change. The change process is one that involves old social practices being displaced through a process influenced by idiosyncratic contingencies (Geels & Schot 2007) and everyday communication is a signal that the dynamic innovation process is being adopted within ‘network[s]’, through ‘social learning’ involving ‘dynamics of power and conflict’ (Leeuwis & Aarts 2011:30). With convergence with Kippax *et al.* (2013) this reiterates that ‘everyday communication among stakeholders is of critical importance for the re-ordering of social relationships and the emergence of space for change in networks’ (Leeuwis & Aarts 2011:33). These processes are often characterised by a ‘certain slowness’ (Cilliers 2006) as people unlearn their existing views or perceptions of a challenge; re-frame the challenge and then begin to learn new ways to tackle it (Rogers, Luton, Biggs, Biggs, Blignaut, Choles, *et al.* 2013). This enabled the overriding aim of the pilots to be articulated: to determine if community communication about HIV alters in ways that reflect a shift in social practices, in turn indicating a possible reduction in the aggregate community viral load. From this platform, the deliverables, or objectives of the pilots became:

1. To clarify if the emergence of new ideas would increase the diversity of prevention and chronic management options available within grassroots communities; and
2. To monitor the emergent implementation strategies and to use everyday communication as an indicator for evaluating the uptake of the initiative within the targeted communities—based on the reality that these pilots would not include scientific measurement of the community viral load.

Based upon this—and any other relevant developments—the conceptual prototype framework will be adapted into an implementation strategy. The monitoring of this work will enable an evaluation of whether the implementation framework can be applied in different contexts and at different scales. The adaptation process will be an iterative process, interdependent with the feedback and inputs of the partner communities.

## 5. Discussion

### 5.1. The common epistemological ‘fault line’ within prevention designs

**Fig. 4.**
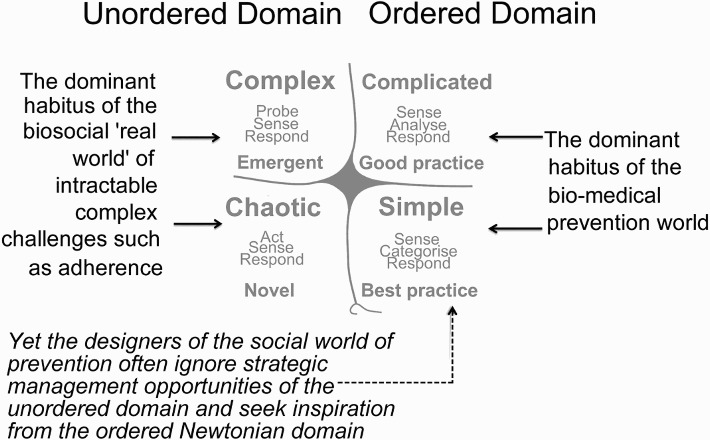
The epistemic fault line that the prototype framework aims to avoid.

Source: Adapted from http://en.wikipedia.org/wiki/Cynefin.

After Adam (2011), we were concerned that the ‘fault line’ is a confusion that primarily relates to epistemology and one that we have deliberately infused into the framework design. It has been argued that biomedical responses to the epidemic are legitimately based upon an ordered way of thinking where the quest for cause and effect is justifiable. However, bio-social responses to the epidemic are often unordered activities; yet—we suggest—the prevailing schema that dominates bio-social responses tends to be incorrectly situated within the parameters of the ordered domain (Baker, Leon & Collins 2011). This is precisely the type of situation that the Cynefin framework was designed to pre-empt: ‘the Cynefin framework can help executives sense which context they are in so that they can not only make better decisions but also avoid the problems that arise when their preferred management style causes them to make mistakes’ (Snowden & Boone 2007:69). This position is outlined in Fig. 4.

Figure 4 provides a normative representation of the ‘habitus’ different agents prefer to occupy. However, in the ‘real world’, it seems that the faultline is rarely bridged. More often than not, the biomedical world becomes frustrated by the unordered uncertainty that ‘culture’ presents during implementation (Nguyen, Bajos, Dubois-Arber, O'Malley & Pirkle 2011) and the bio-social world tends to design and evaluate social prevention efforts that are implicitly under-written by the constraints associated with the Newtonian, ordered domain. Typically the Newtonian rules are ‘grounded in positivism where practices, characteristics and attributes are abstracted from context and fixed into place as variables, and then correlated through probabilistic statistics’ (Adam 2011:4)—which is an inappropriate management response to an unordered, complex situation.

An alternative, more synergistic, position is to overcome the fault line by applying a multi-ontology implementation approach—underwritten by a ‘middle-ground’ focus on both agency and structure. This is acknowledged in the prototype by including the Cynefin framework so that appropriate management techniques can be applied to both the ordered and unordered challenges. The practical benefit of opening the prevention frame to both ordered and unordered responses that is inclusive of both agency and structure is that it opens the armamentarium into a relatively untrodden, qualitatively unique domain of diverse possibilities—thus options—to disrupt conditions that enable the virus to flourish.

### 5.2. Overview

We have described a journey that was designed to broaden the knowledge of HIV transmission and introduced a novel implementation strategy designed for rural communities. As the pilots unfold the collective learning will shape its future identity.

**Fig. 5.**
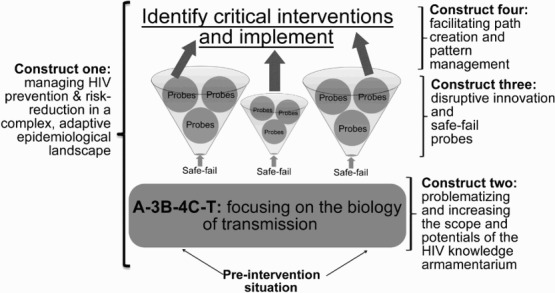
The ambition—dynamic HIV-Conversant Communities, situated within, and influencing the trajectory of, a complex adaptive epidemiological landscape in ways that promote well-being and disrupt the ability of the virus to replicate and/or be transmitted from one body to another.

Source: Authors’ contribution.

We consider this to be an intervention that incorporates up to date ordered, scientific information about HIV transmission which has the potential to be incorporated into the unordered and complex life-worlds and—ultimately—the social practices of people within diverse communities. We imported four constructs—mostly from outside of the realm of traditional HIV discourse—and this is represented in Fig. 5.

The design was an iterative process that now incorporates the following:

1. *Re-framing the challenge: HIV as a complex adaptive epidemiological landscape.* The epidemic represents a ‘long wave event’ and can be described as a ‘complex adaptive epidemiological landscape’ (Burman *et al*., forthcoming) that can be manipulated, thus managed in ways that reduce the aggregate community viral load. This involves a dynamic form of management that is inclusive of both ordered and unordered response opportunities and the prototype framework embraces this by way of a multi-ontology design;
2. *Construct one: managing HIV-prevention in a complex, adaptive landscape.* The history of prevention represents a variable ebb and flow—intimately tied to biomedical gains—and tends to be biased towards the ‘ordered domain’ of the Cynefin framework. By situating responses exclusively within the ordered domain occludes the critical interrogation of alternative opportunities situated within the ‘unordered domain’. The universal prototype design has the potential to promote a diversity of localised responses that make sense to particular target groups in different contexts, thereby responding to the NSP's call for innovation and UNAIDS’ call to involve communities in the ambition to ‘end AIDS by 2030’;
3. *Construct two: problematising and increasing the scope and potentials of the HIV knowledge armamentarium.* An educational framework that goes beyond the limitations of individualised, instructive prevention—such as that of the ABC genre—by focusing on knowledge about the science of transmission was introduced as a basis for a more dynamic management frame. The reason for this is that obeisance to an individualised, rational mental model of prevention restricts the prevention portfolio at a time when increased diversity of ideas for the ‘personalisation’ of *acquiring*, *facilitating* or *transmitting* the virus are required;
4. *Construct three: disruptive innovation and safe-fail probes.* Disruptive innovation was used as a guiding metaphor to emphasise that the greater the diversity of ideas, the greater the likelihood that new social practices can be propagated. In a practical sense, combining disruptive innovation with safe-fail learning opportunities provisions participants with opportunities for ‘dreaming big and includ[ing] the whole human family’ by ‘shak[ing] off outdated modes of thinking’ about their relationship/s with the epidemic in ways that do not jeopardise the well-being of the host organisation or community (UNAIDS 2014:296);
5. *Construct four: Facilitating path creation and pattern management.* The application of path creation enables both a retrospective and prospective frame that can expand the perspectives for co-managing the emergence of ideas and activities that surface during the safe-fail component of the interventions. It also enables a holistic, middle-ground monitoring and evaluation design component that is inclusive of both agency and structure. Pattern management bolsters the path creation process by reinforcing activities that are deemed important to achieve the desired end-state and also offers opportunities to dampen, or obstruct, activities that are deemed to be counter-productive;
6. *Ambition of the pilots:* to transform the prototype framework into a viable implementation strategy in partnership with rural communities.

## 6. Conclusion

We have mapped out a transdisciplinary journey that has focused on expanding the HIV prevention armamentarium in South Africa from an ontologically restrictive, individualised discourse towards a multi-ontology conceptualisation that aims for synergy between the ordered and unordered epistemological responses to HIV prevention and risk-reduction.

The overall ambition is threefold. First, to facilitate the development of skills which will enable people to better manage, thus negotiate the epidemiological landscape—thereby potentially reducing the aggregate community viral load, contributing to ‘ending AIDS by 2030’. Second, to develop a better understanding of the processes involved so that the approach can be taken to scale if appropriate. Third, it was also designed to begin the process of opening a ‘chronic situation’ schema.

We introduced four concepts that include: managing HIV-prevention in a complex, adaptive landscape; problematising and increasing the scope and potentials of the HIV knowledge armamentarium; disruptive innovation and safe-fail probes and the facilitation of path creations and implementation based on pattern management.

The framework design process was iterative and we expect that it will develop further as the pilots unfold. The overriding concern during the design process was that there is now such a rich kaleidoscope of complex dynamics embedded within the epidemiological landscape—which will inevitably influence the future trajectory of the epidemic—that it is essential to include them within future prevention strategies. This required a radical shift to comprehensively embrace the complexity of community landscapes—including their local dynamics and the relevance of local environmental conditions—so that a broad portfolio of responses can be generated and developed into functional strategies to reduce the aggregate viral load. The framework represents a universal design that has the potentials to be applied in multiple contexts because the safe-fail probe component is hard wired to incorporate localised diversities into the safe-fail intervention strategies.

Evidence from the forthcoming pilots will provide indicators of the efficacy of this style of transdisciplinary management approach for contributing to reducing the overall burden of HIV in South Africa and possibly open opportunities for imagining new strategies for managing other chronic conditions in rural contexts.
